# Multiple instance ensembling for paranasal anomaly classification in the maxillary sinus

**DOI:** 10.1007/s11548-023-02990-3

**Published:** 2023-07-21

**Authors:** Debayan Bhattacharya, Finn Behrendt, Benjamin Tobias Becker, Dirk Beyersdorff, Elina Petersen, Marvin Petersen, Bastian Cheng, Dennis Eggert, Christian Betz, Anna Sophie Hoffmann, Alexander Schlaefer

**Affiliations:** 1 Institute of Medical Technology and Intelligent Systems, Technische Universitaet Hamburg, Hamburg, Germany; 2 Department of Otorhinolaryngology, Head and Neck Surgery and Oncology, University Medical Center Hamburg-Eppendorf, Hamburg, Germany; 3 Clinic and Polyclinic for Diagnostic and Interventional Radiology and Nuclear Medicine, University Medical Center Hamburg-Eppendorf, Hamburg, Germany; 4 Population Health Research Department, University Heart and Vascular Center, University Medical Center Hamburg-Eppendorf, Hamburg, Germany; 5 Clinic and Polyclinic for Neurology, University Medical Center Hamburg-Eppendorf, Hamburg, Germany

**Keywords:** Paranasal anomaly, Maxillary sinus, CNN, Classification

## Abstract

****Purpose**:**

Paranasal anomalies are commonly discovered during routine radiological screenings and can present with a wide range of morphological features. This diversity can make it difficult for convolutional neural networks (CNNs) to accurately classify these anomalies, especially when working with limited datasets. Additionally, current approaches to paranasal anomaly classification are constrained to identifying a single anomaly at a time. These challenges necessitate the need for further research and development in this area.

****Methods**:**

We investigate the feasibility of using a 3D convolutional neural network (CNN) to classify healthy maxillary sinuses (MS) and MS with polyps or cysts. The task of accurately localizing the relevant MS volume within larger head and neck Magnetic Resonance Imaging (MRI) scans can be difficult, but we develop a strategy which includes the use of a novel sampling technique that not only effectively localizes the relevant MS volume, but also increases the size of the training dataset and improves classification results. Additionally, we employ a Multiple Instance Ensembling (MIE) prediction method to further boost classification performance.

****Results**:**

With sampling and MIE, we observe that there is consistent improvement in classification performance of all 3D ResNet and 3D DenseNet architecture with an average AUPRC percentage increase of 21.86 ± 11.92% and 4.27 ± 5.04% by sampling and 28.86 ± 12.80% and 9.85 ± 4.02% by sampling and MIE, respectively.

****Conclusion**:**

Sampling and MIE can be effective techniques to improve the generalizability of CNNs for paranasal anomaly classification. We demonstrate the feasibility of classifying anomalies in the MS. We propose a data enlarging strategy through sampling alongside a novel MIE strategy that proves to be beneficial for paranasal anomaly classification in the MS.

## Introduction

Paranasal sinuses, located within specific bones, are prone to pathologies like retention cysts and polyps [1–3]. These anomalies, although often incidental, pose challenges for healthcare professionals, as they are unrelated to the patient’s primary clinical indications [4]. Multiple studies emphasize the importance of understanding and addressing the prevalence of these paranasal anomalies in the general population [5–9].

Accurate diagnosis of paranasal inflammations is crucial for effective patient care, with medical professionals relying on CT and MRI scans to assess the extent of these conditions in the head and neck area [10]. 3D information is essential for identifying paranasal anomalies correctly, as misdiagnosis can lead to patient distress and increased healthcare costs [11]. A retrospective study found misdiagnoses of inverted papillomas and malignant tumors as nasal polyps in a significant percentage of cases [12]. Deep learning (DL) methods offer potential for improving diagnostic accuracy and reducing clinicians’ workload, but the highly variable anatomy of paranasal sinuses necessitates cautious consideration when applying these techniques for reliable and accurate diagnoses [13].

DL technologies have shown significant advancements in anomaly detection, particularly in computer vision [14] and medical imaging analysis [15]. CNNs have proven effective in paranasal pathology screening, sinusitis classification, and tumor subtype differentiation. Existing studies typically follow a two-stage approach of localizing sinuses and then classifying them. For instance, one study cropped X-rays and classified anomalies [16], but failed to classify left and right MS anomalies separately. Another study segmented Computed Tomography (CT) images and classified anomalies [17], necessitating pixel-level annotations for localization. A different approach involved using a CNN to detect key slices in CT images containing MS volumes and then classifying MS anomalies [18]. However, two-stage methods relying on specialized annotations for localization and classification pose challenges in terms of increased clinician workload and limited generalization to diverse datasets.

Our proposed end-to-end approach is a non-DL solution for localizing MS volumes and a DL method for classifying MS anomalies. By employing a unique localization strategy using Gaussian sampling of centroid coordinates, we significantly expand the dataset and extract multiple overlapping instances of the MS. Leveraging a 3D CNN in our MIE prediction approach, we achieve boosted classification performance for these volumes. Through rigorous experimentation involving popular DenseNet and ResNet architectures, we ascertain the optimal MS volume size for achieving superior classification performance. Our comprehensive pipeline proves to be advantageous for accurate MS anomaly classification, providing a promising alternative to DL-based methods.

## Methods

*Dataset*: as part of the Hamburg City Health Study (HCHS) [19], cMRIs of participants (45–74 years) were recorded for neuroradiological assessment. These scans were obtained at the University Medical Center Hamburg-Eppendorf and feature fluid attenuated inversion recovery (FLAIR) sequences in the NIfTI format. The dataset comprises 299 patients, with 174 exhibiting healthy left and right MS and 125 exhibiting at least one MS having a polyp or cyst pathology. The diagnoses were confirmed by two ear, nose, and throat (ENT) surgeons and one ENT specialized radiologist. The anomalies under consideration in this study include polyps and cysts. MS exhibiting these anomalies are grouped into “anomalous” class and MS without these anomalies are grouped into “normal” class.

**Fig. 1:**
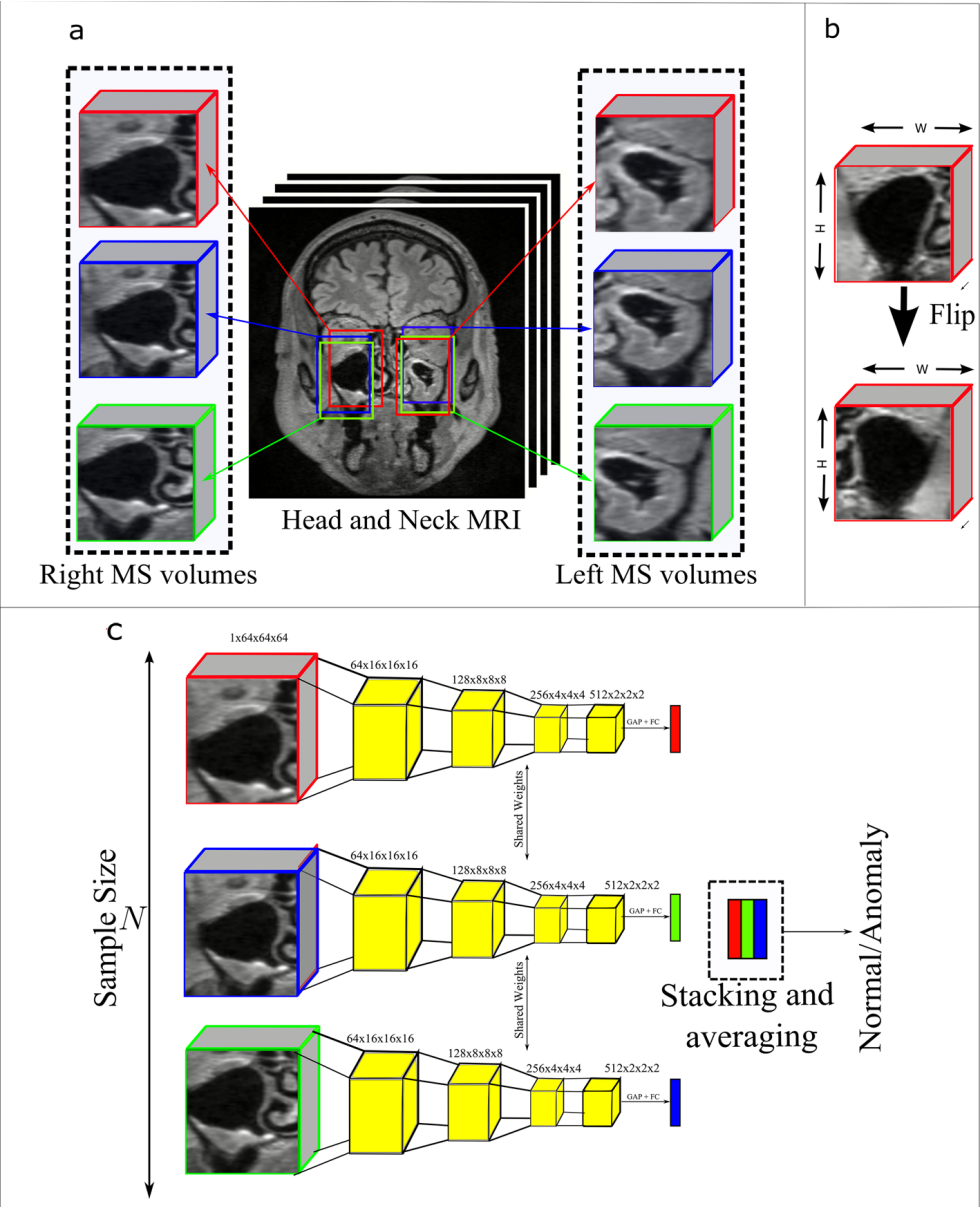
**a** Illustration of our MS volume extraction strategy showing 3 MS volumes for left and right MS each. **b** Flipping of the coronal plane of the right MS. **c** Illustration of our MIE prediction strategy used during inference. GAP denotes Global Average Pooling and FC denotes Fully Connected Layer

*Dataset preprocessing and MS volume extraction*: each MRI in the study has a resolution of 173x319x319 voxels, with a voxel size of 0.53 $\times$ 0.75 $\times$ 0.75 mm. To ensure consistency across all of the head and neck MRI scans in our study, we apply a process of rigid registration using Dipy library [20]. This involves selecting one MRI as a fixed volume and registering other MRIs with respect to the fixed volume.

**Fig. 2:**
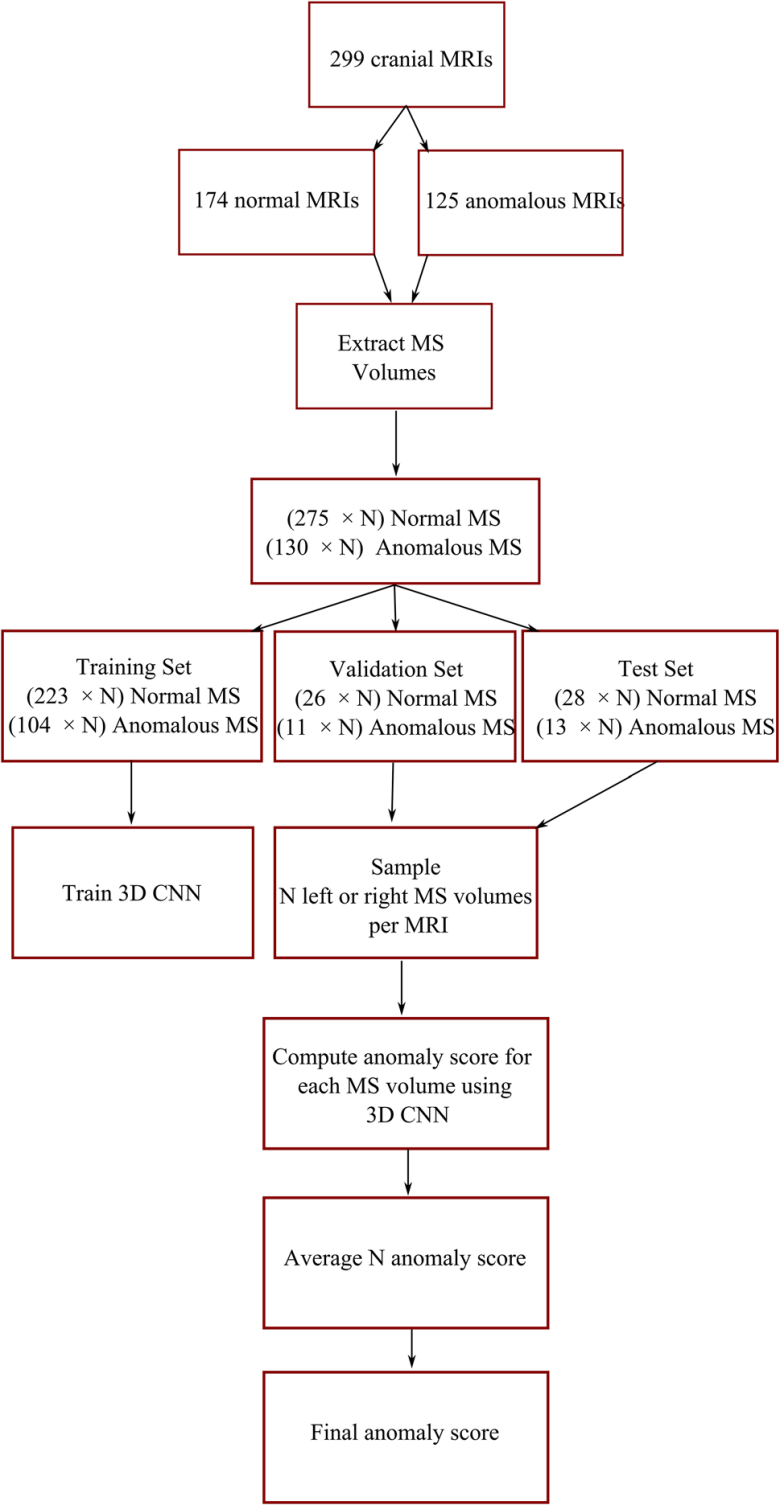
Illustration of our data processing pipeline

To increase the size of the dataset and be able to use multiple instances of MS volumes for our ensemble prediction, we extracted multiple MS volumes of left and right MS from individual head and neck MRI scans. This was done by manually recording the centroid locations of the left and right MS of 20 patients, and using these coordinates to compute the mean and standard deviation of the centroid locations. These values are denoted as $\mu (x),\mu (y),\mu (z)$ and $\sigma (x),\sigma (y),\sigma (z)$ for the mean and standard deviation, respectively. We then initialize Gaussian distributions - $\mathcal {N}(\mu (x),\sigma ^{2}(x))$,$\mathcal {N}(\mu (y),\sigma ^{2}(y))$,$\mathcal {N}(\mu (z),\sigma ^{2}(z))$—and use these distributions to sample centroid locations for MS volumes in the head and neck MRI. It is worth noting that the mean and standard deviation of the left and right MS volumes are different, resulting in a total of six Gaussian distributions in practice. In practice, for the left MS the *x*, *y* and *z* $\mu$ are 75, 231 and 121 mm and $\sigma$ are 1.47, 1.56 and 1.76 mm, respectively. Correspondingly for the right MS, the x, y and z $\mu$ are 149, 232 and 118 mm and $\sigma$ are 1.90, 1.66 and 6.47 mm respectively. We sample $N$ left MS volumes and $N$ right MS from each head and neck MRI where $N$ is the sample size. For our experiments, $N \in \{1,5,10,15,20\}$. An illustration of our sampling method is shown in Fig. 1a. We extract MS volumes of multiple sizes namely, 25 $\times$ 25 $\times$ 25, 30 $\times$ 30 $\times$ 30, 35 $\times$ 35 $\times$ 35, 40 $\times$ 40 $\times$ 40, 45 $\times$ 45 $\times$ 45. The extracted MS volumes are finally resampled to a resolution of 64 $\times$ 64 $\times$ $\times$ 64 for the 3D CNN. To make the right and left MS appear more symmetrical, we horizontally flip the coronal planes of the right MS to give it the appearance of the left MS volume. Figure 2 illustrates our data processing pipeline.

*Training, validation and test splits*: if we sample with $N=1$, we end up with 327, 37 and 41 MS volumes in the training, validation and test set, respectively. The training validation and test set size increase by a factor of $N$ with respect to the sample size $N$. 32% of the MS volumes in the training, validation and test sets are anomalous MS volumes. We perform threefold cross-validation experiments with all the methods.

*Implementation details* We implement a 3D CNN using ResNet18 [21] with four stages of 3D residual blocks (channel dimensions 64, 128, 256, 512). Our models are trained for 100 epochs with a batch size of 16, a learning rate of 0.0001, and Adam optimization. If the validation loss did not improve for 5 epochs, the learning rate is reduced by a factor of 10. We use PyTorch and PyTorch Lightning to build our models.

*DL method*: to classify the MS volume into normal or anomaly class, we consider multiple 3D ResNet [21]^1^ and 3D DenseNet[22] architectures.^2^ Let us denote the classifier as $f(\cdot )$ and the MRIs as $X \in R^{H \times W \times D}$. From each MRI, we extract $N$ left MS volumes and $N$ right MS volumes. Altogether, we extract 2$N$ MS volumes from $X \in R^{H \times W \times D}$. Let us denote the MS volumes as $x \in R^{P \times P \times P}$. Here, $P$ denotes the size of the MS volume such that $P \in \{25,30,35,40,45\}$. Further, our labels $y \in \{0,1\}$ represent normal and anomaly class. The anomaly class is the positive class for our use-case. As a baseline, we consider 3DResNet models that do not use our MIE strategy for inferring on the test set.

*Multiple instance ensemble prediction strategy*: let us denote the extracted MS volumes from a single MRI $x_{i} \in R^{P \times P \times P}$ where $i$ denotes the $i-th$ MS volume extracted from either the left or right MS area of the MRI. When making a prediction, we average the softmax scores of classifier $f(.)$ from the multiple MS volumes $x_{i}$. Formally,

$$\begin{aligned} \hat{y} = \frac{1}{N}\sum _{i=1}^{N} softmax(f(x_{i})) \end{aligned}$$

## Results

### Effect of sampling size

We plot the mean and standard deviation of the Area Under Precision Recall Curve (AUPRC) and F1 score. Both these metrics are useful especially in imbalanced scenarios which is our case. From Table 1, we observe that with the increase in the sample size $N$, we get a consistent increase in all the reported metrics until $N=15$ after which we get a decrease in all the metrics. Further, for all the cases, we see that using MIE strategy is beneficial for MS anomaly classification and leads to boost in classification metrics.

### Comparison with state-of-the-art 3D CNN architectures

We investigate the benefits of sampling and ensembling techniques on 3D CNN architectures for medical imaging classification. Specifically, we examine their impact on 3D DenseNet and 3D ResNets used in various medical imaging tasks [23–28]. Using $N=15$, we conduct an ablation study, training the CNNs with sampled data but inferring with a single MS volume. We observe that, for ResNets, the AUPRC decreases as architecture complexity increases, but with $N$=15 and MIE enabled, the decrease is minimal. Sampling and sampling with MIE consistently improve performance for both ResNets and DenseNets, resulting in percentage increases of 21.86 ± 11.92% and 4.27 ± 5.04% (sampling) and 28.86 ± 12.80% and 9.85 ± 4.02% (sampling + MIE) in AUPRC, respectively

### Effect of sampling type

**Fig. 3:**
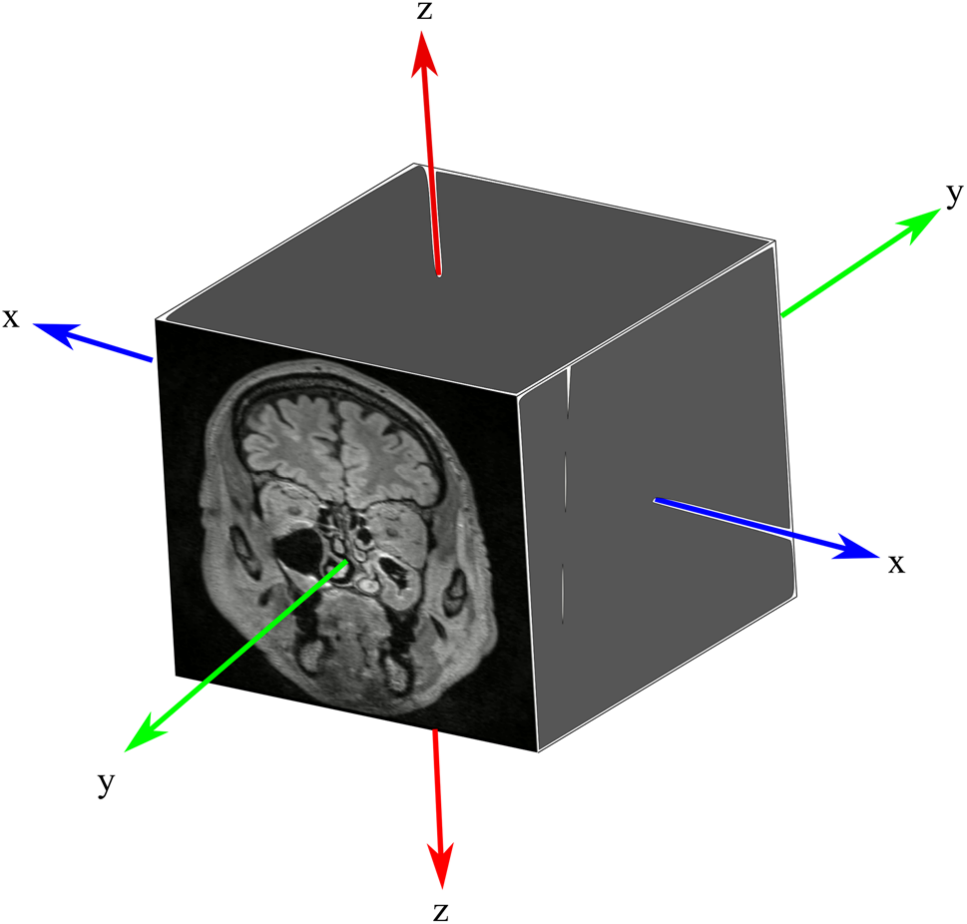
*x*, *y* and *z* axis for an MRI

In our methods, we adopt a Gaussian distribution to model centroid locations and sample random centroids for extracting corresponding MS volumes. However, an alternative approach is to extract equidistant centroids, which allows us to investigate potential advantages of random sampling. For this experiment, we consider the x, y, and z coordinates of the centroid to lie on lines starting at $\mu (x)$ - $\sigma (x)$, $\mu (y)$ - $\sigma (y)$ and $\mu (z)$ - $\sigma (z)$, respectively, and ending at $\mu (x)$ + $\sigma (x)$, $\mu (y)$ + $\sigma (y)$ and $\mu (z)$ + $\sigma (z)$. Figure 3 illustrates the relationship between these axes and the MRI image from which the coordinates are sampled. Utilizing these lines, we sample $N$ equidistant centroid locations, while also exploring variations such as fixing one or two coordinates at their means. Referred to as *equidistant sampling*, we compare this strategy and its variants against the random sampling method we propose. Table 3 presents our findings, demonstrating the most advantageous sampling from the *z*-axis for classification. Equidistant sampling from all axes yields an AUPRC of 0.89 ± 0.03, while equidistant sampling from x and y axes with a constant z coordinate results in an AUPRC of 0.82 ± 0.16, showcasing an 8.18% difference. Similarly, fixing the *y*-coordinate incurs a 1.12% AUPRC decrease, while maintaining a constant *z*-coordinate exhibits no AUPRC decrease. These results suggest varying importance in sampling MS centroid coordinates from each axis, with random sampling proving to be the most effective approach.

**Table 1:** Result of our experiments

*N*	MIE	AUPRC	F1
1		0.80 ± 0.12	0.70 ± 0.13
5		0.85 ± 0.03	0.77 ± 0.10
5	$\checkmark$	0.87 ± 0.04	0.76 ± 0.10
10		0.85 ± 0.04	0.75 ± 0.08
10	$\checkmark$	0.89 ± 0.05	0.79 ± 0.10
15		0.88 ± 0.07	0.81 ± 0.12
15	$\checkmark$	**0**.**92** ± **0**.**06**	**0**.**85** ± **0**.**09**
20		0.87 ± 0.04	0.77 ± 0.05
20	$\checkmark$	0.91 ± 0.02	0.78 ± 0.07

Patch size $P$ = 35 for all the experiments and 3D ResNet18 architecture used

The bold signifies the highest/best metric in each column of a table

**Table 2:** Result of our experiments

CNN	N	MIE	AUPRC	F1
3D ResNet18	1		0.80 ± 0.12	0.70 ± 0.13
3D ResNet18	15		0.88 ± 0.07	0.81 ± 0.12
3D ResNet18	15	$\checkmark$	0.92 ± 0.06	0.85 ± 0.09
3D ResNet50	1		0.72 ± 0.13	0.59 ± 0.19
3D ResNet50	15		0.82 ± 0.11	0.71 ± 0.19
3D ResNet50	15	$\checkmark$	0.85 ± 0.07	0.74 ± 0.13
3D ResNet101	1		0.73 ± 0.10	0.59 ± 0.04
3D ResNet101	15		0.85 ± 0.04	0.69 ± 0.10
3D ResNet101	15	$\checkmark$	0.90 ± 0.06	0.79 ± 0.14
3D ResNet152	1		0.66 ± 0.06	0.57 ± 0.08
3D ResNet152	15		0.83 ± 0.07	0.76 ± 0.11
3D ResNet152	15	$\checkmark$	0.89 ± 0.05	0.80 ± 0.08
3D ResNet200	1		0.60 ± 0.21	0.45 ± 0.39
3D ResNet200	15		0.86 ± 0.05	0.79 ± 0.10
3D ResNet200	15	$\checkmark$	0.90 ± 0.04	0.83 ± 0.07
3D DenseNet121	1		0.86 ± 0.11	0.80 ± 0.07
3D DenseNet121	15		0.86 ± 0.10	0.81 ± 0.06
3D DenseNet121	15	$\checkmark$	0.92 ± 0.05	0.83 ± 0.12
3D DenseNet169	1		0.81 ± 0.09	0.76 ± 0.11
3D DenseNet169	15		0.91 ± 0.05	0.82 ± 0.04
3D DenseNet169	15	$\checkmark$	0.94 ± 0.03	0.86 ± 0.09
3D DenseNet201	1		0.88 ± 0.07	0.72 ± 0.07
3D DenseNet201	15		0.88 ± 0.04	0.72 ± 0.08
3D DenseNet201	15	$\checkmark$	0.93 ± 0.06	0.78 ± 0.07
3D DenseNet264	1		0.84 ± 0.09	0.81 ± 0.07
3D DenseNet264	15		0.88 ± 0.05	0.82 ± 0.12
3D DenseNet264	15	$\checkmark$	0.93 ± 0.01	0.85 ± 0.09

Patch size $P$ = 35 for all the experiments

**Table 3:** Experiment on sampling strategy

*x*	*y*	*z*	AUPRC	F1
$\mu (x)$	$\mu (y)$ ± $\sigma (y)$	$\mu (z)$ ± $\sigma (z)$	0.89 ± 0.03	0.74 ± 0.03
$\mu (x)$ ± $\sigma (x)$	$\mu (y)$	$\mu (z)$ ± $\sigma (z)$	0.88 ± 0.04	0.77 ± 0.04
$\mu (x)$ ± $\sigma (x)$	$\mu (y)$ ± $\sigma (y)$	$\mu (z)$	0.82 ± 0.16	0.73 ± 0.19
$\mu (x)$	$\mu (y)$	$\mu (z)$ ± $\sigma (z)$	0.89 ± 0.03	0.77 ± 0.04
$\mu (x)$	$\mu (y)$ ± $\sigma (y)$	$\mu (z)$	0.85 ± 0.08	0.68 ± 0.18
$\mu (x)$ ± $\sigma (x)$	$\mu (y)$	$\mu (z)$	0.85 ± 0.04	0.75 ± 0.03
$\mu (x)$ ± $\sigma (x)$	$\mu (y)$ ± $\sigma (y)$	$\mu (z)$ ± $\sigma (z)$	0.89 ± 0.03	0.79 ± 0.06
$\mathcal {N}(\mu (x),\sigma ^{2}(x))$	$\mathcal {N}(\mu (y),\sigma ^{2}(y))$	$\mathcal {N}(\mu (z),\sigma ^{2}(z))$	**0.92 ± 0.07**	**0.85 ± 0.09**

Patch size $P$ = 35 for all the experiments. $\mu$  ±  $\sigma$ represents equidistant sampling of $N$ points from a line starting at $\mu$ - $\sigma$ and ending at $\mu$ + $\sigma$. $\mathcal {N}(\mu ,\sigma ^{2})$ represents random sampling of points from a Gaussian distribution parameterized by $\mu$ and $\sigma$. 3D ResNet18 architecture used

### Effect of patch size

**Fig. 4:**
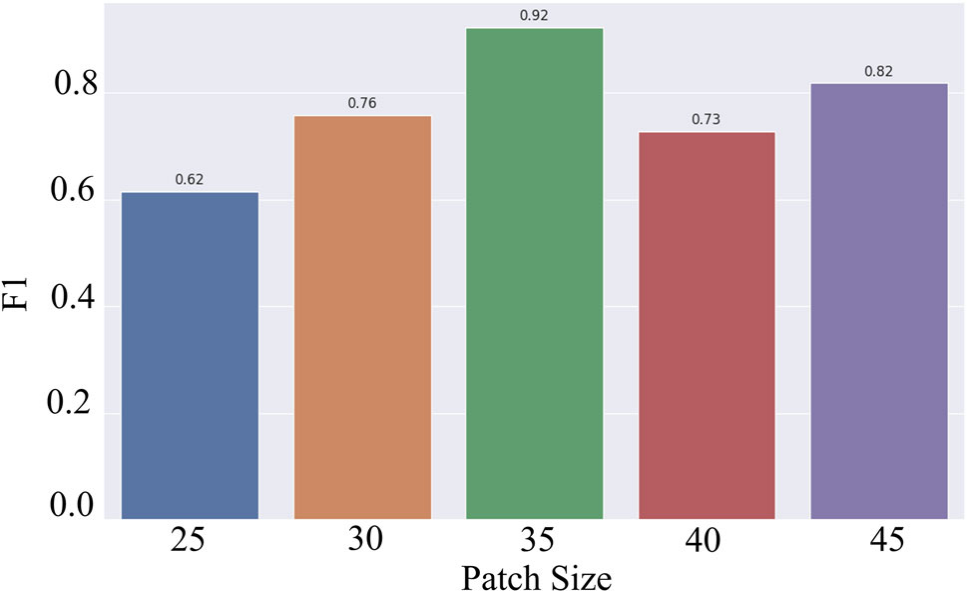
F1 scores vs patch size $P$

Further, looking at Fig. 4, we can see the influence of MS volume size to the parnasal classification task. Note, we set $N=15$ for this experiment and use our multiple instance ensemble prediction strategy. This highlights that patch size plays an important role in boosting the paranasal anomaly classification performance. Our experiments indicate that the optimal patch size for our dataset is $P = 35$.

**Fig. 5:**
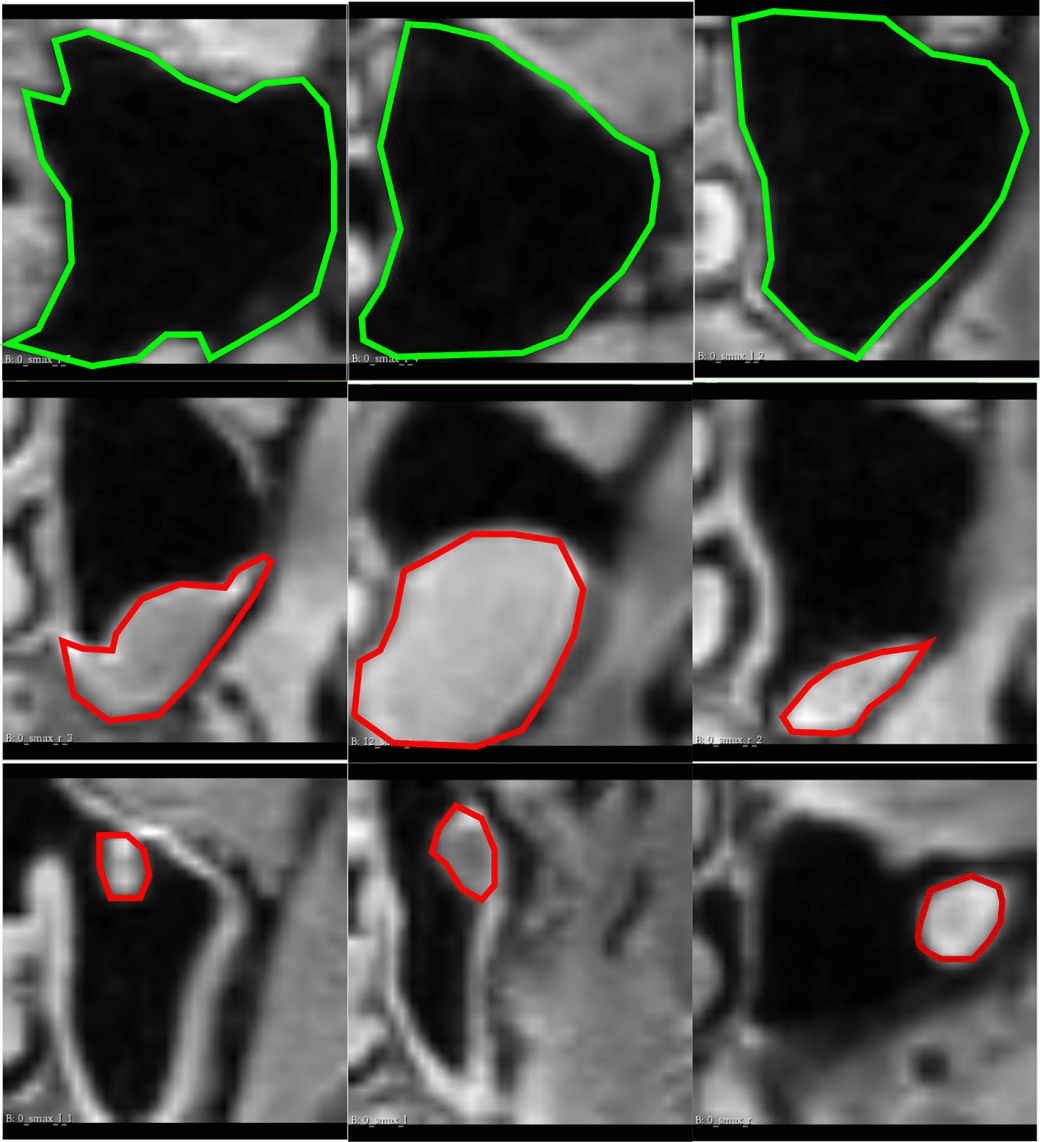
The coronal planes of the sampled MS volumes. The green contours in the first row represent normal MS anatomy. The red contours enclose masses that represent cysts and polyps in the second and third row, respectively, demonstrating the variety of appearances and morphological variations of these anomalies within the MS

## Discussion

Clinicians classifying paranasal anomalies face the burdensome task of manually searching for MS-containing slices in MRI sequences and then diagnosing, resulting in time-consuming and fatiguing analysis. Additionally, the task of classifying paranasal anomalies is challenging due to the morphological variation of the maxillary sinus as well as the polyp and cyst anomalies inhabiting the sinuses. This can be seen in Fig. 5. Previous approaches [18] used a 2-stage CNN pipeline, learning key MS slices first using a CNN and then classifying anomalies with another CNN [18] or learning to segment the maxillary sinus and then classifying the anomaly [17], but these methods 2 stage CNN pipeline makes it dependent on datasets hindering generalization. To overcome this limitation, we propose a method that extracts multiple MS volumes without DL, using a CNN only once to compute the final anomaly score for each MS. This streamlined approach reduces dataset dependency and facilitates broader applicability to other modalities.

**Fig. 6:**
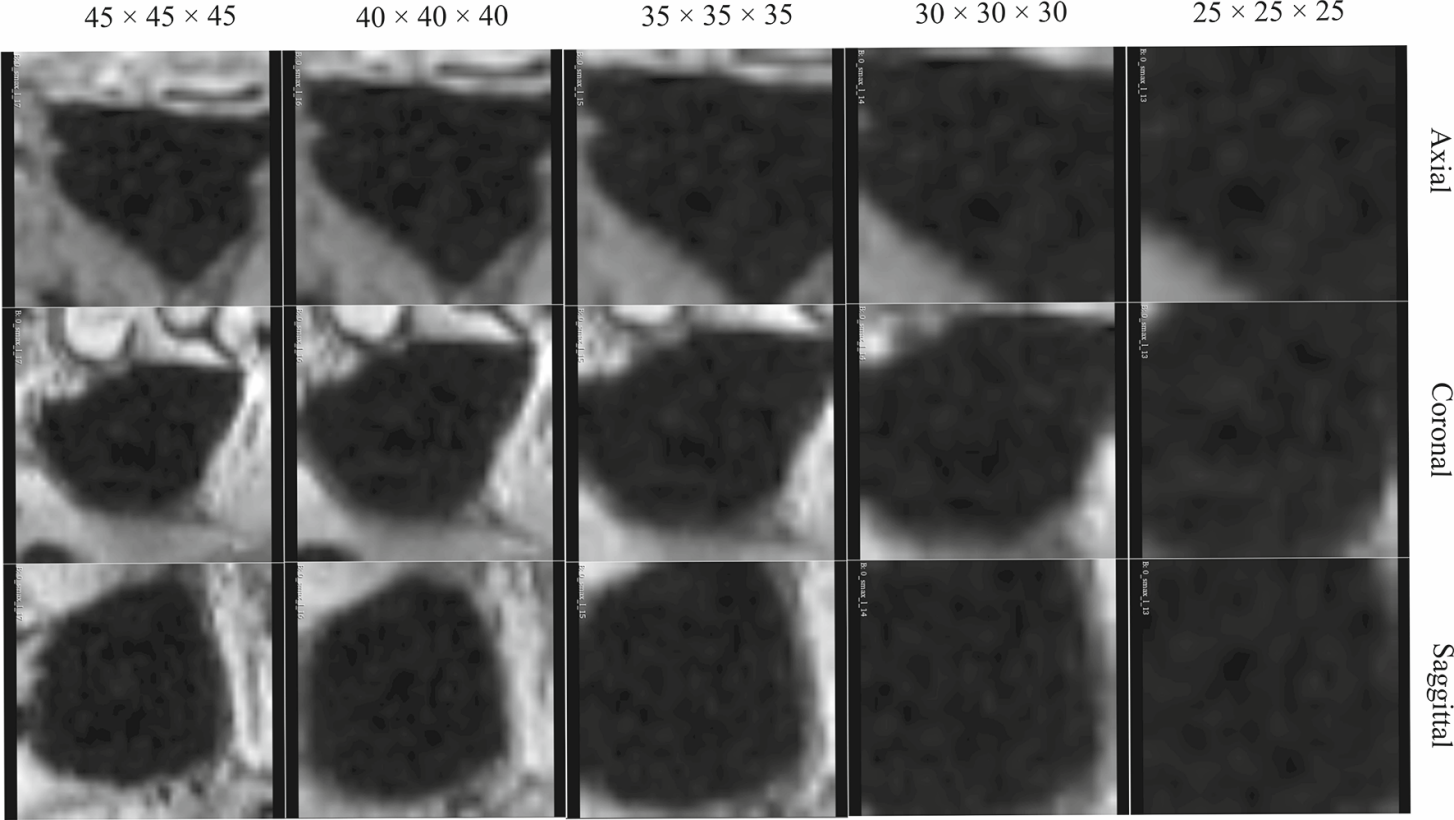
Slices from the axial, coronal and saggital slices of extracted healthy MS volume with different patch sizes

As seen in Table 1, increasing the sample size $N$ improves classification metrics, but a sample size of 20 exhibits a lower F1 score compared to 15, possibly due to overfitting caused by redundant volumes. Thus, careful selection of the appropriate sample size is crucial for optimal performance.

We compared different CNN architectures in Table 2 and found that sampling and MIE are beneficial for our classification task. The advantages of MIE and sampling are more prominent in ResNet architectures compared to DenseNet. We hypothesize that the multiple skip connections in a dense block [29] contribute to improved gradient flow and optimization in the $N$ = 1 sampling scenario. Our method consistently increases AUPRC, with sampling + MIE showing higher efficacy than only sampling and no sampling, demonstrating the effectiveness of our approach.

We compared random sampling and equidistant sampling’s impact on classification performance in Table 3. Random sampling yielded better results, likely due to the diverse multi-scale volumes obtained through randomization. Equidistant sampling along the *z*-axis significantly improved classification performance, possibly due to higher *z*-coordinate $\sigma$, resulting in greater spatial offsets. This increased diversity in sampled volumes facilitated better feature learning and classification performance. Incorporating multi-scale volumes from different z-axis positions enhanced the ability of our 3D CNN to identify patterns, improving overall performance.

Additionally, using an ensemble strategy that averages the scores from multiple instances of the MS leads to a further improvement in classification metrics. The improvement in our classification metrics can be attributed to the incorporation of implicit test-time augmentation during inference on the test set. By sampling multiple overlapping MS volumes, we have MS volumes which have transnational offsets with respect to one another, resulting in better performance. These findings demonstrate the utility of our proposed method for the classification of paranasal anomalies in the MS.

The size of the extracted MS volume is critical for accurate classification. Small volumes may miss important details, while large volumes include irrelevant information as can be seen in Fig. 6. Our evaluation of different sizes (25 $\times$ 25 $\times$ 25 to 45 $\times$ 45 $\times$ 45) found that 35 $\times$ 35 $\times$ 35 yielded the highest F1 score. This suggests that small volumes miss anomalies, while larger volumes include unnecessary structures. Careful selection of the patch size is crucial for optimal performance.

## Conclusion

We propose a DL approach for classifying maxillary sinus anomalies. Our method employs multiple instance ensemble prediction and a sampling strategy to improve classification performance on available dataset. We investigate the optimal sample size and patch size trade-off. Although further improvements are needed for real-world clinical use, our work offers a promising solution for maxillary sinus anomaly classification with DL.

## Footnotes

1 https://github.com/kenshohara/3D-ResNets-PyTorch/blob/master/models/resnet.py.

2 https://github.com/kenshohara/3D-ResNets-PyTorch/blob/master/models/densenet.py.
